# Kidney Injury in Critically Ill Patients with COVID-19 – From Pathophysiological Mechanisms to a Personalized Therapeutic Model

**DOI:** 10.2478/jccm-2023-0023

**Published:** 2023-07-31

**Authors:** Cosmin Balan, Tudor Ciuhodaru, Serban-Ion Bubenek-Turconi

**Affiliations:** Prof. Dr. C. C. Iliescu Emergency Cardiovascular Diseases Institute, Bucharest, Romania; Prof. Dr. Nicolae Oblu Emergency Clinical Hospital, Iași, Romania; Carol Davila University of Medicine and Pharmacy, Bucharest, Romania

**Keywords:** acute kidney injury, COVID-19, renal replacement therapy, acute respiratory distress syndrome, personalized cardiocirculatory support

## Abstract

Acute kidney injury is a common complication of COVID-19, frequently fuelled by a complex interplay of factors. These include tubular injury and three primary drivers of cardiocirculatory instability: heart-lung interaction abnormalities, myocardial damage, and disturbances in fluid balance. Further complicating this dynamic, renal vulnerability to a “second-hit” injury, like a SARS-CoV-2 infection, is heightened by advanced age, chronic kidney disease, cardiovascular diseases, and diabetes mellitus. Moreover, the influence of chronic treatment protocols, which may constrain the compensatory intrarenal hemodynamic mechanisms, warrants equal consideration. COVID-19-associated acute kidney injury not only escalates mortality rates but also significantly affects long-term kidney function recovery, particularly in severe instances. Thus, the imperative lies in developing and applying therapeutic strategies capable of warding off acute kidney injury and decelerating the transition into chronic kidney disease after an acute event. This narrative review aims to proffer a flexible diagnostic and therapeutic strategy that recognizes the multi-faceted nature of COVID-19-associated acute kidney injury in critically ill patients and underlines the crucial role of a tailored, overarching hemodynamic and respiratory framework in managing this complex clinical condition.

## Introduction

The first cases of pneumonia caused by the novel coronavirus SARS-CoV-2 were officially recognized by the World Health Organization (WHO) on December 31, 2019. According to the latest WHO reports, as of 14 June 2023, there have been approximately 768 million confirmed cases of infected individuals globally, with 6.9 million deaths [1]. COVID-19 infection presents a wide spectrum of clinical severity, ranging from asymptomatic to self-limiting pulmonary forms and culminating in acute respiratory distress syndrome (ARDS), multiorgan dysfunction, and death[2]. Conceptually, such clinical heterogeneity results from a unique host-virus interaction combined with the therapeutic strategies employed [3,4].

In SARS-CoV-2 infection, renal involvement, which was initially neglected, has been reappraised. It is now confirmed that, in combination with cardiocirculatory alterations, SARS-CoV-2 may invade renal tubular cells, leading to acute kidney injury (AKI) associated with COVID-19 (COVID-AKI), which proportionally escalates morbidity and mortality rates with the progression of the disease stage [5,6,7]. In a study by McNicholas et al., AKI incidence and outcomes in COVID-19 ARDS (CARDS) patients were compared to a non-CARDS cohort from the pre-pandemic LUNG-SAFE (Large observational study to understand the global impact of severe acute respiratory failure) study [8,9]. Findings revealed that COVID-19 ARDS patients had a lower early AKI incidence and cardiovascular SOFA score, yet a higher mortality rate. Furthermore, it is evident that COVID-AKI is linked with unfavorable long-term kidney function recovery, underscoring the critical need for the development and implementation of therapeutic strategies that can deter the onset or progression into chronic kidney disease following an initial AKI event [10].

## Epidemiology

Numerous studies, underscored by two meta-analyses, have demonstrated the frequent occurrence of COVID-AKI, especially among critically ill patients, evidencing an average incidence rate of 11% (8–17%) among hospitalized patients, and up to 23% (14–35%) for those requiring admission to the intensive care unit (ICU) [11,12]. Building upon this, a recent study by Bubenek-Turconi et al. on a Romanian cohort of 9058 ICU-admitted COVID-19 patients unveiled a 24.1% prevalence of COVID-AKI[13]. Noteworthy, in the subset of the very elderly patients, the same authors found that COVID-AKI emerged as the second most prevalent complication (27%), surpassed only by ARDS with an incidence of 33% [14].

The incidence varies significantly between studies, with the highest reported rates seen in North America and Europe [13,15,16,17,18,19,20,21,22,23,24,25,26,27,28,29](see ***Table 1***). This variation is largely attributed to several factors, including the inconsistent use of the KDIGO staging for AKI, irregular measurement or reporting of baseline serum creatinine, ambiguity concerning recent renal function history, differences in study populations, failure to differentiate between de novo AKI and acute-on-chronic kidney disease, reporting bias, and timing of data collection. Importantly, a key discrepancy arose from the absence of an operational definition of AKI in many studies. When this methodological tool was included, stages according to the Kidney Disease Improving Global Outcomes (KDIGO) classification were not consistently reported, thereby rendering a retrospective epidemiological analysis impractical on most occasions [7,15,22,29]. By meeting these two minimum requirements (i.e., KDIGO definition and KDIGO staging), Hirsch et al. precisely examined COVID-AKI in a retrospective study on a cohort of 5449 patients infected with SARS-CoV-2[17] (see ***Table 1***). Renal impairment was rapidly progressive, with 37.3% of patients meeting KDIGO criteria within 24 hours of admission, and mortality increased proportionally with the stage of AKI. Respiratory failure had significant renal consequences when it called for invasive positive pressure mechanical ventilation (IPPV) (adjusted OR for AKI development: 10.7 [6.81 – 16.7]). Thus, among patients who required IPPV, 89.7% subsequently developed AKI compared to only 21.7% of those who remained unventilated. The majority of stage 3 AKI cases (518 out of 619 [83.6%]), as well as most patients requiring renal replacement therapy (RRT) (276 out of 285 [96.8%]), were found among those who received IPPV. Globally, RRT was instituted in 9 out of 4259 (0.2%) non-ventilated patients compared to 276 out of 1190 (23.2%) patients exposed to IPPV. AKI events remained concentrated around the time of IPPV initiation, with 52.2% of cases confirmed within 24 hours of intubation. Alongside IPPV, the multivariate analysis revealed other independent risk factors, with the second most important factor being the use of vasoactive support (adjusted OR: 4.53 [2.88 – 7.13]). ***Table 2*** provides an exhaustive list of potential risk factors for the development of COVID-AKI, as outlined in references [11,30,31].

**Table 1.** AKI incidence in patients with COVID-19 disease

**Author and Reference**	**Location**	**Period**	**Definition**	**Patients no.**	**Critically ill no.**	**COVID-AKI no. (%)**	**COVID-AKI in ICU no. (%)**	**RRT no. (%)**
Bubenek-Turconi [13]	Romania	25.03.2020–26.03.2021	KDIGO	9058	9058	2183 (24.1)	2183 (24.1)	453 (5)
Huang [15]	Wuhan	16.12.2019–02.01.2020	KDIGO	41	13	3 (7.31)	3 (23.08)	3 (7.31)
Richardson [16]	New York	01.03.2020–04.04.2020	KDIGO	5700/2351^#^	373	523 (22.2)	NR	81 (3.4)
Hirsch [17]	New York	01.03.2020–05.04.2020	KDIGO + all stages	5449	1395	1993 (36.6)	1060 (76)	285 (5.2)
Gupta [18]	USA	04.03.2020–04.04.2020	KDIGO stage 2/3	2215	2215	952 (43)	952 (43)	443 (20)
Mohamed [19]	Louisiana	01.03.2020–31.03.2020	KDIGO	575	173	161 (28)	105 (61)	89 (15.5)
Schaubroeck [20]	Belgium	01.02.2020–31.01.2021	KDIGO + all stages	1286	1286	1094 (85.1)	1094 (85.1)	126 (9.8)
Sullivan [21]	United Kingdom	17.01.2020–5.12.2020	KDIGO + all stages	85687	NR	13000 (31.5)	NR	2198 (2.6%)
Wang [22]	Wuhan	01.01.2020–03.02.2020	KDIGO	138	36	5 (3.62)	3 (8.33)	2 (1.45)
Guan [23]	China	11.12.2019–29.01.2020	KDIGO	1099	173	12 (1.09)	6 (3.47)	9 (0.82)
Cao [24]	Wuhan	03.01.2020–01.02.2020	KDIGO	102	18	20 (19.61)	8 (44.44)	6 (5.88)
Zhang [25]	Wuhan	02.01.2020–10.02.2020	KDIGO	221	55	10 (4.52)	8 (14.55)	5 (2.26)
Xu [26]	China	01.01.2020–20.02.2020	NR	355	71	56 (15.77)	21(29.58)	NR
Li Z [27]	China	06.01.2020–21.02.2020	KDIGO	193	65	55 (28.5)	43(66.15)	7 (3.63)
Zheng [28]	Hangzhou	22.01.2020–05.03.2020	KDIGO	34	34	7 (20.59)	7 (20.59)	5 (14.71)
Arentz [29]	Seattle	20.02.2020–05.03.2020	KDIGO	21	21	4 (19.05)	4 (19.05)	NR

CVD, cardiovascular disease; DM, diabetes mellitus; ESRD, end-stage renal disease; HTN, hypertension; ICU, intensive care unit; KDIGO, Kidney Disease: Improving Global Outcomes; No., number; NR, not reported; RRT, renal replacement therapy;

# number of patients (i.e., 2351 out of 5700) for whom COVID-AKI incidence was reported and RRT was initiated.

**Table 2.** Potential risk factors associated with COVID-AKI

**Socio-demographic risk factors**	**Risk factors at admission**	**Post-admission risk factors**
Advanced age (> 70 years)	Elevated viremia	Nephrotoxins (e.g., contrast agents)
Diabetes mellitus	Leukocytosis and lymphopenia	Vasopressors
Hypertension	Increased levels of ferritin, CRP, and D-dimers	Mechanical ventilation
Congestive heart failure	Hypovolemia/dehydration	Hypovolemia
Obesity	Multiorgan involvement	Hypervolemia
Chronic kidney disease	Rhabdomyolysis	Metabolic disturbances (e.g., hyperglycemia)
Immunosuppression	Exposure to ACE inhibitors, ARBs, and NSAIDs	Fluid imbalances (e.g., use of hydroxyethyl starch, increased chloride levels)

ACEI = angiotensin converting enzyme inhibitor; ARB = angiotensin receptor blocker; CRP = C reactive protein; HES = hydroxyethyl starch; NSAID = non-steroidal anti-inflammatory drug.

AKI, already recognized as an unfavorable prognostic factor in the general population of critically ill patients, was associated with increased mortality in SARS-CoV-2 infection as well[7,26,32,33]. However, the severity of AKI may play a crucial role as Cheng et al. suggested that only KDIGO stages 2 and 3 increased the risk of death (HR: 3.53 [1.5–8.27]) [7].

Renal impairment is also reflected in other indicators beyond serum creatinine. Their prognostic and therapeutic impact needs further evaluation. Proteinuria was frequently reported in SARS-CoV-2 infections, with an incidence from 7% to 63% of cases [5,7,27,34]. Hematuria was found in 26.7% of patients and, along with proteinuria, was associated with an increased rate of in-hospital mortality [7]. On rare occasions, reported sporadically in African-origin patients admitted for COVID-19, proteinuria was massive and accompanied a rare variant of focal segmental glomerulosclerosis known as “collapsing” glomerulopathy [35,36,37]. A correct interpretation of these results requires consideration of the following aspects: 1) pre-admission proteinuria values were not known, and the patients included in these studies often exhibited pre-existing risk factors (e.g., hypertension, diabetes mellitus, chronic kidney disease) that could have contributed to the observed post-admission proteinuria; 2) the association with mortality may indicate both the severity of SARS-CoV-2 infection and the severity of underlying comorbidities; 3) although podocyte injuries can result from direct viral aggression, in the case of individuals of African origin, the APOL1 genotype cannot be excluded as it represents an equivalent contributing factor in the genesis of “collapsing” glomerulopathy [38]; 4) in the context of AKI, quantifying proteinuria other than through direct measurement risks overestimating protein excretion over a 24-hour period [39].

## Pathophysiology

**Fig. 1.**
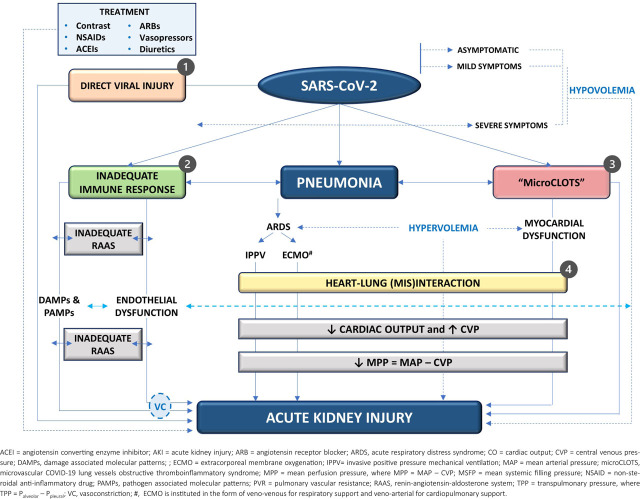
**Pathophysiology of AKI in COVID-19.** AKI arises from multiple intricated mechanisms, including 1) glomerulo-tubular injuries secondary to potentially direct viral cytopathic effects, 2) an inadequate immune response, initially localized to the lungs and later becoming systemic, 3) a ubiquitous process of thrombotic microangiopathy referred to as “microCLOTS,” and 4) a complex heart-lung interaction that requires active and individualized therapeutic intervention. Endothelial dysfunction is an all-pervasive driver of organ dysfunction. There is inadequate activation of RAAS, leading to both immediate and long-term renal consequences such as glomerular dysfunction, inflammation, fibrosis, and vasoconstriction. The initiation of IPPV has hemodynamic repercussions dependent on lung mechanics: 1) in the L subphenotype (i.e., normal lung elastance), the gradient that ensures venous return (MSFP - CVP) is reduced, mimicking hypovolemia; 2) in the H subphenotype (i.e., increased lung elastance), an increased TPP along with other pulmonary and extrapulmonary factors (e.g., hypoxemia, hypercapnia, microthrombosis in pulmonary and cardiac capillaries, hypervolemia), contribute to the development of pulmonary artery hypertension and acute cor pulmonale. A reduced MPP is the end result of all hemodynamic derangements. This may involve a decrease in MAP with or without a decrease in CO, an increase in CVP, or both. Medications can have aggravating consequences. An adequate hemodynamic and respiratory support should avoid fluid overload, reduce vasopressor doses, and optimize MPP and systemic tissue perfusion.

Still under evaluation, the pathophysiological mechanisms implicated in the development of COVID-AKI exhibit dynamic and intricate interconnections, with certain variables capable of contributing to multiple causal pathways[11,30,31,40]. These mechanisms can manifest either non-specifically or specifically in response to SARS-CoV-2 infection, serving as the foundation for the therapeutic approach delineated below (see ***Figure 1***).

### Non-specific pathophysiological mechanisms

A series of factors such as advanced age, chronic kidney disease, cardiovascular diseases, and diabetes mellitus are associated with immune senescence and chronic inflammation that results in increased renal vulnerability to a “second-hit” injury such as the SARS-CoV-2 infection [41]. Equal consideration should be given to the chronic treatment that may limit the compensatory intrarenal hemodynamic mechanisms [31,42].

Imaging studies are essential in critically ill patients, and the use of contrast agents becomes inevitable in this already at-risk population. Their contribution in the genesis of AKI has been reviewed, with the latest evidence downgrading their role from a determinant one (i.e., CIAKI - contrast-induced acute kidney injury) to, at most, a contributory one (i.e., CAAKI - contrast-associated acute kidney injury) [43,44,45,46,47,48,49]. A recently published multi-site propensity-matched analysis led by Ehmann et al. found that contrast administration was safe and inconsequential even among patients with pre-existing AKI [50]. Consequently, the preventive strategy for contrast-induced nephropathy has been simplified and overlaps with the optimization of hemodynamics per se in critically ill patients, and, in the absence of other harmless alternatives (e.g., MRI, ultrasound), the diagnostic benefit takes precedence over the risk of AKI [43,51].

IPPV and cardiocirculatory failure conduce to an augmented sympathetic adrenergic tone and activation of the renin-angiotensin-aldosterone system (RAAS). These two systems have been proposed as the drivers of a generalized shock-induced endotheliopathy that ultimately involves a myriad of interorgan crosstalk signaling factors, including cytokines, growth factors, and damage-associated molecular patterns (DAMPs) [52]. In the pathogenesis of CARDS, AKI may arise from an intricate interplay between the kidneys and the lungs, as evidenced by renopulmonary crosstalk [53,54,55]. It is noteworthy that various other tissues, beyond the lungs, can also contribute to the release of DAMPs, which further fuel AKI development [56,57,58].

Cardiocirculatory failure in COVID-19 is propelled by multiple mechanisms that can act singularly or combined to disrupt renal inflow and outflow dynamics: 1) hypovolemia, 2) cardiac dysfunction, and 3) vasoplegia and peripheral vascular maldistribution.

In COVID-19, insensible water losses are common at admission to the intensive care unit (ICU) and directly proportional to clinical severity [15,23]. Volume depletion requires immediate therapeutic intervention to avoid a prerenal insult and to improve tissue oxygen supply. Conversely, fluid overload is equally harmful and, when associated with AKI, indicates an increased risk of mortality (adjusted RR 2.63 [1.30–5.30])[59,60].

It is universally accepted that judicious fluid administration, especially in the context of CARDS, breaks down to two macrohemodynamic principles: 1) fluid responsiveness and 2) fluid tolerance [61,62,63]. Recently, it has been demonstrated that macrohemodynamic fluid responsiveness does not guarantee microhemodynamic responsiveness, hence the importance of monitoring the microcirculatory perfusion [64]. Failure to consider this aspect can jeopardize the integrity of the glycocalyx, thereby exacerbating fluid therapy-associated complications[65,66]. Therefore, intensivists face the challenging task of finding a balance between two functional microhemodynamic extremes while simultaneously respecting these two macrohemodynamic principles: 1) limited convective flow that corresponds to an insufficient microcirculatory fluid filling associated with a low density of functional capillaries and 2) limited diffusion due to excessive fluid that leads to increased intercapillary distances and reduced density of functional capillaries [67]. The resolution of this dilemma remains a debated topic and is open for further research. The most recent solution proposes a cumulative parameter, the tissue red blood cell perfusion index (tRBCp), which incorporates both convective and diffusive components of tissue perfusion[68]. Although its quantification is automated and feasible at the bedside, additional studies are still needed to justify and describe its implementation in current practice.

Ultimately, the type of fluid administered can directly influence AKI. High chloride content and hydroxyethyl starch-based fluids were associated with an increased incidence of AKI[69,70]. Similarly, dextrans and gelatin were associated with an increased risk of bleeding, AKI, and mortality [71,72]. Therefore, current guidelines recommend balanced crystalloids over other fluids [30,73]. However, some recent trials and meta-analyses have indicated no discernible clinical advantage of balanced solutions over the utilization of 0.9% saline solutions [74,75]. Consequently, the rational approach to fluid selection aligns more with a strategy that tailors to the patient's biochemical profile and electrolyte imbalances, rather than a generic, one-size-fits-all strategy [76]. Additionally, based on recent trial data from patients with severe sepsis or septic shock, the administration of albumin has been suggested to potentially be associated with a trend towards reduced mortality [77,78].

Cardiorenal syndromes, commonly observed in COVID-19, can arise due to primary cardiac impairments such as myocarditis, in situ micro- and macro-immunothrombosis, pulmonary embolism, or the intricate interplay between the heart and lungs associated with IPPV [31,73]. Controversially, there was a proposition that the mechanical characteristics of CARDS encompass a wide spectrum that includes a subphenotype characterized by preserved lung elastance (referred to as L subphenotype) and another one characterized by increased lung elastance (referred to as H subphenotype) [79,80,81,82,83,84,85]. Similarly, Filippini et al. used latent class analysis to split CARDS subphenotypes based on their recruitment potential [86]. Additionally, compared to all-cause ARDS, Chiumello et al. showed that CARDS patients exhibited higher compliance and lung gas volume for the same oxygenation parameters, lower recruitment potential and higher blood flow redistribution [87]. The two subphenotypic extremes could very well represent different stages of disease progression. Ferrando et al. proposed that COVID-19 calls for a flexible and adaptable IPPV strategy, as the lung mechanics shift from an L phenotype found in the early stages to an H phenotype found in the late stages of COVID-19 [88]. The most recent ARDS guidelines incorporate and merge all recommendations for both non-CARDS and CARDS cases [89]. Regardless of the underlying cause, the alterations in pulmonary mechanical constants are accompanied by corresponding changes in the hemodynamic mechanisms associated with adequate renal perfusion. Overlooking this principle by employing high positive end-expiratory pressure indiscriminately was linked to a fivefold increase in the risk of COVID-AKI and elevated mortality, as evidenced in an observational study by Ottolina et al [90]. Consequently, the provision of circulatory support may necessitate an approach based on the physiological characteristics specific to the different stages of CARDS (i.e., early vs. late period) [91]. At all times, maintaining an optimal mean perfusion pressure and preventing central venous congestion is of utmost importance, as their breach has been associated with a heightened occurrence of AKI [92,93,94,95,96]. To this end, hemodynamic monitoring is crucial and must be personalized, combined, and comprehensive, incorporating both ultrasound and transpulmonary thermodilution methods, to characterize the functional cardiac reserve, and vigilantly avoid common pitfalls associated with the presence of acute cor pulmonale or low tidal ventilation [97,98] (see ***Table 3***).

**Table 3.** CARDS phenotyping – a mechanistic overview.

**Criterion**	**CARDS subphenotype**	
	**L subphenotype**	**H subphenotype**
Pulmonary mechanics	E_L_ and ECW are normal EELV is normal Normal strain and stress at TV 6–8ml/kg IBW	E_L_ is increased and ECW is normal EELV is reduced Increased strain and stress at TV 6–8ml/kg IBW
Computer Tomography	Aerated Ground glass Normal weight	Dependent atelectasis Condensations Increased weight
Histopathologic substrate	microCLOTS	Diffuse alveolar damage
Gas exchange abnormality	V/Q mismatch Decreased fluid tolerance	Shunt Severely decreased fluid tolerance
Positive pressure transmission *P_pleural_ = P_alveolar_ × (E_CW_/E_T_)*	Mainly in the pleural space P_pleural_ increases, so then CVP increases	Mainly transpulmonary Alveolar pressure increases, so then TPP increases, TPP = P_alveolar_ - P_pleural_
Cardiac effects	RV preload is reduced Mimicking hypovolemia	RV afterload is increased Risking acute cor pulmonale
Renal effects	Decreased arterial flow Decreased MPP	Decreased arterial flow Decreased MPP Venous congestion
Respiratory strategy	Low recruitment potential Avoid open lung approach PP responsiveness is low	High recruitment potential Individualized open lung approach PP responsiveness is high
Hemodynamic strategy	Prevent fluid overload. Optimize RV preload	Reduce lung water. Optimize RV afterload
Hemodynamic monitoring	Ultrasound TPTD PPV/SVV: useful for fluid management.	Ultrasound TPTD PPV/SVV: less useful, increased rate of false negatives if used with VT < 8ml/kg IBW or of false positives if acute cor pulmonale ensues. A VT challenge helps discriminate the false negatives. Cardiac ultrasound helps discriminate the false positives.

CARDS, COVID-19 induced acute respiratory distress syndrome; CVP, central venous pressure; E_L_, lung elastance; E_CW_, chest wall elastance; E_T_, total elastance where E_T_ is E_L_ + E_CW_; EELV, end expiratory lung volume; IBW, ideal body weight; MAP, mean arterial pressure; MPP, mean perfusion pressure where MPP = MAP– CVP; PP, prone position; PPV/SVV, pulse pressure variation/stroke volume variation; RV, right ventricle; TPP, transpulmonary pressure where TPP is P_alveolar_ – P_pleural_; TPTD, transpulmonary thermodilution; TV, tidal volume; V/Q, ventilation/perfusion.

### Specific Pathophysiological Mechanisms of SARS-CoV-2

The kidney fulfills all the molecular prerequisites for direct involvement by SARS-CoV-2. This theoretical renal tropism is based on the following main aspects: 1) angiotensin-converting enzyme 2 (ACE-2), the receptor for SARS-CoV-2, is expressed in podocytes and the proximal tubule and 2) transmembrane serine protease 2, responsible for the cleavage of the Spike (S) protein of the novel coronavirus, is predominantly detectable in tubular cells and to a lesser extent in glomerular cells[99,100]. Additionally, CD147 has recently been described as a potential receptor for the S protein, and its abundance in the proximal tubule provides further evidence supporting the hypothesis of direct renal viral invasion [101,102].

Nevertheless, despite the elevated theoretical likelihood, the direct viral cytopathic effects on the kidney in COVID-19 have largely remained uncertain, given the conflicting histopathological findings. Several post-mortem studies demonstrated the presence of viral particles in tubular and podocyte cells under electron microscopy as well as SARS-CoV-2 viral RNA in glomerular cells [103,104,105]. The apparent clinical consequences of this renal tropism were an increased incidence of AKI and a higher risk of premature death [106]. On the contrary, real-time PCR analysis of renal autopsy samples did not detect SARS-CoV-2, raising doubts about the existence of COVID-19 nephropathy [107].

The underlying biological mechanisms of this potential direct viral invasion of renal tissue were explored by Dudoignon et al. who reported increased direct and indirect markers of RAAS activation in a cohort of 51 patients, particularly among those who developed AKI[108]. However, the specificity of this association with SARS-CoV-2 is difficult to assess due to the absence of: 1) a control arm consisting of AKI patients without COVID-19 and 2) a cytopathological diagnosis of direct viral aggression in patients infected with the novel coronavirus. In this regard, Puskarich et al. conducted a randomized clinical trial involving 13 hospitals to investigate the impact of SARS-CoV-2 on RAAS homeostasis. The study findings revealed that losartan, in contrast to the anticipated outcome, did not improve oxygenation after 7 days and resulted in a decreased number of vasopressor-free days compared to the placebo group[109].

Most recently, Perego et al. conducted a single-centre retrospective study that analyzed post-mortem kidney samples from critically-ill patients with COVID-19[110]. AKI was prevalent, affecting 55.8% of the population, and molecular biology analyses were performed on the renal tissues of 46% of the patients, detecting SARS-CoV-2 in only 20% of the samples, with no discernible difference between the AKI and non-AKI groups. Noteworthy, no evidence of direct viral damage, such as interstitial inflammatory infiltrate, was identified, suggesting that renal injury may be the consequence of multifactorial influences, with hemodynamic instability as a paramount contributor. In line with these data, Paranjpe et al., through the analysis of clinical and proteomic data, posited that while both acute and long-term kidney dysfunction associated with COVID-19 correspond with markers of tubular dysfunction, AKI is primarily driven by a multifaceted process encompassing hemodynamic instability and myocardial damage [111].

In general, while certain mechanisms of SARS-CoV-2 may contribute to the development of AKI, they do not constitute the primary focus of the targeted treatment strategies.

## Treatment

COVID-AKI treatment involves a strategy of prevention and optimization of cardiopulmonary and metabolic parameters, which largely overlaps with the non-differentiated therapy of critically ill patients. The recommendations below align with the consensus of the Acute Disease Quality Initiative (ADQI) working group [30].

### Prevention and optimization of cardiopulmonary and metabolic parameters

General preventive measures aim to avoid or mitigate the impact of risk factors, such as 1) contrast agents used in imaging studies, 2) antibiotics with renal excretion and metabolism, and 3) medication that may limit the compensatory intrarenal hemodynamic mechanisms such as the non-steroidal anti-inflammatory drugs, angiotensin-converting enzyme inhibitors, and angiotensin-receptor blockers (see ***Table 4***).

**Table 4.** Preventive measures in COVID-AKI

**Intervention**	**Argument**	**Recommendation**
Renal function	Staging AKI and assessing clinical risk are epidemiological imperatives with crucial therapeutic implications.	Recommend the use of serum creatinine and urine output for monitoring renal function, paying attention to limitations of both parameters. (Level of evidence: 1B)
Hemodynamic profiling	Inadequate tissue perfusion contributes to the worsening of organ dysfunction (e.g., kidney, lung, liver, and heart).	Recommend an individualized hemodynamic strategy based on dynamic and quantitative indices of cardiovascular evaluation. (Level of evidence: 1B)
Fluids	Fluid composition has systemic consequences, including renal. High chloride content was associated with an increased incidence of AKI, and the use of hydroxyethyl starch derivatives in sepsis is contraindicated.	Recommend the use of balanced crystalloids for initial volume resuscitation in at-risk patients or those who develop COVID-AKI, in the absence of other specific indications. (Level of evidence: 1A)
Glycemic control	Insulin resistance and hypercatabolism are frequently encountered in patients with COVID-19.	Suggest the use of an intensive glycemic control strategy. (Level of evidence: 2C)
Nephrotoxins	Various nephrotoxins are commonly prescribed to patients with COVID-19.	Recommend limiting exposure to nephrotoxic medications and vigilant monitoring when they cannot be avoided. (Level of evidence: 1B)
Contrast agents	The relevance of contrast agent toxicity is uncertain.	Recommend optimizing intravascular volume as the only preventive measure. (Level of evidence: 1A)
Mechanical ventilation	Increased intrathoracic pressure results in: 1) elevated central venous pressures and peripheral venous congestion; 2) sympathetic adrenergic and renin-angiotensin-aldosterone system activation; 3) mechanical disadvantage, particularly for the right ventricle; 4) renal, hepatic, and splanchnic cross-talk.	Suggest the use of a protective ventilatory strategy for both the lungs and the right ventricle, individualized and continuously tailored to the patient's real-time physiology. (Level of evidence: 2C)

Metabolic priorities in AKI coincide with those of critically ill patients, focusing primarily on two components: 1) intensive glycemic control and 2) nutritional support, with particular attention to protein intake.

The principles of cardiopulmonary support have been discussed in the subsection dedicated to non-specific pathophysiological mechanisms contributing to COVID-AKI development. These principles are universally applicable, with the key message that mean arterial pressure (MAP) and central venous pressure (CVP) are equally important in ensuring renal perfusion. An increase in CVP is associated with decreased glomerular perfusion, AKI, and death [112,113]. Legrand et al. reported a direct proportional relationship between CVP and the prevalence of AKI. Additionally, the same authors demonstrated no association between AKI (new or persistent) and classic macrohemodynamic parameters such as MAP, cardiac output (CO), and mixed venous oxygen saturation [114]. Another study revealed the ability of venous congestion to stratify the risk of AKI development and confirmed the lack of predictive value of CO in this regard [115]. In recent studies, there has been a growing focus on both poles of renal perfusion, namely MAP (i.e., renal preload) and CVP (i.e., renal afterload), demonstrating that the time-weighted average of mean perfusion pressure (MPP, calculated as MAP - CVP) is associated with an elevated risk of AKI and renal adverse events [116]. These findings underscore the importance of considering cardiocirculatory optimization strategies, as represented in ***Table 4*** and adapted from the insights provided in references [11,30,31].

**Fig. 2.**
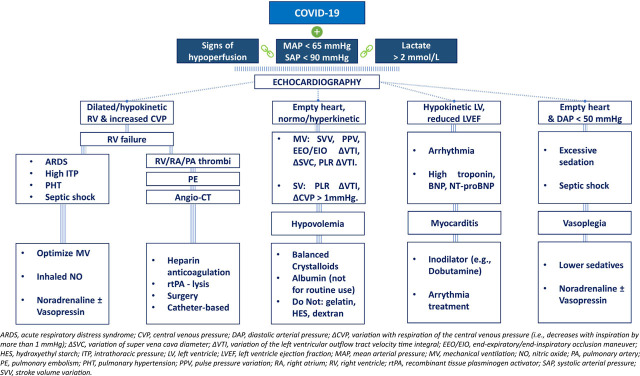
**Echocardiography as a tool to diagnose, monitor and treat cardiocirculatory collapse**.

Ultrasound imaging has emerged as an invaluable tool in this context, offering a swift and non-invasive means of assessing diverse cardiocirculatory and pulmonary parameters [117]. Consequently, amid the COVID-19 pandemic, we devised an algorithm that relies on echocardiographic evaluation, specifically designed to promptly diagnose and address hemodynamic instability and shock in affected patients (***see Figure 2***).

### Renal replacement therapy

The COVID-19 pandemic has presented unprecedented challenges for healthcare teams and patients alike. When there is improved access to RRT, the implementation of a coordinated local response by medical staff can significantly reduce the mortality rate. Efficient and fair allocation of limited medical resources can be achieved through various measures, including optimizing the modality and indications for RRT, adopting appropriate anticoagulation strategies, and carefully determining the dosage of RRT [31].

Assuming that human, technical, and material resources are not limiting factors, the guidelines for implementing RRT in COVID-19 align closely with the initiation of RRT in critically ill patients [16,17]. Nonetheless, it should be acknowledged that anticoagulation treatment protocols may require a more flexible implementation in individuals infected with SARS-CoV-2. Moreover, given the diminished fluid tolerance intrinsic to COVID-AKI, the process of fluid removal mandates an individualized approach, congruent with each patient's unique fluid tolerance [118]. Just as in resuscitation, the adoption of a functional hemodynamic algorithm during fluid removal could be superior to a generic approach [119,120,121,122,123,124,125]. This tailored strategy aims to prevent both under-resuscitation scenarios, characterized by residual vascular and extravascular congestion, and over-resuscitation scenarios, which may result in low cardiac output and hypotension (see ***Table 5***).

**Table 5.** Recommendations for the good clinical practice of RRT

**RRT Component**	**Management**
Indication	When metabolic byproducts (e.g., hyperkalemia, acidosis, hypervolemia) exceed renal clearance. An individualized approach that should consider the decreased fluid tolerance observed in patients with severe forms of COVID-19.
Modality	Selection of RRT technique depends on the metabolic and hemodynamic priorities of the patient, as well as on the local expertise and resources. CRRT benefit hemodynamically unstable or fluid overloaded patients. Reduced tolerance to intercompartmental fluid shifts favors the use of CRRT. IHD may be useful in stable hemodynamic patients with progressively favorable outcomes.
Dose	CRRT: effluent rate of 25–30 ml/kg/h. IHD: ≥ 3 sessions/week, alternating days. Adjustment of effluent doses based on individual metabolic needs. Correction of effluent doses based on periods of circuit clotting and transportation outside the ICU. To protect the filter, avoid filtration fractions greater than 20%.
Anticoagulation	Adjusted to coagulation status. RCA: initial dose of 4% trisodium citrate set at 3.5 mmol/L and post-filter Ca^2+^ at 0.25–0.35 mmol/L. HNF: initial dose set at 10–15 IU/kg/h, with a target aPTT of 60–90 seconds. LMWH: initial dose set at 3.5 mg/h, with a target residual anti-Xa activity of 0.25–0.35 IU/ml.
Vascular access	Ultrasound guidance reduces costs and complications. First choice: right internal jugular vein; avoid subclavian access.
Fluid removal	Functional hemodynamic monitoring is essential for optimizing fluid removal rate. In the most basic functional hemodynamic model, the concurrent monitoring of CO, CVP, and MAP is essential. In this model, the ideal removal rate seeks to preserve stable CO and MAP levels while decreasing CVP, all without requiring an escalation of vasoactive support. Sustaining removal rates above 1.75 ml/kg/hour without a hemodynamic feedback loop may worsen hemodynamics.

*CO = cardiac output; CRRT = continuous renal replacement therapy; CVP = central venous pressure; ICU = intensive care unit; IHD = intermittent hemodialysis; LMWH = low-molecular weight heparin; MAP = mean arterial pressure; RCA = regional citrate anticoagulation; RRT = renal replacement therapy; UFH = unfractionated heparin.*

## Conclusion

COVID-AKI is a prevalent condition associated with increased morbidity and mortality. The pathophysiological mechanisms contributing to COVID-AKI, apart from the direct viral cytopathic effect, overlap with those involved in non-viral AKI. As a result, the strategies for prevention, hemodynamic and metabolic optimization, as well as the protocol for initiating RRT, show concurrence between COVID-AKI and non-viral AKI. However, the distinctive feature of COVID-AKI lies in the prothrombotic potential specific to COVID-19, warranting an individualized approach to anticoagulation that judiciously balances each patient's risks of thrombosis and bleeding. Moreover, given the inflammatory context associated with SARS-CoV-2 infection, as well as its propensity to result in severe CARDS with or without cardiocirculatory failure, consideration should be given to the implementation of complementary extracorporeal cytokine adsorption techniques and various forms of extracorporeal life support.
